# ZnO-Functionalized Cotton Textiles with Enhanced Antibacterial Activity, Moisture Management, and Wear Comfort

**DOI:** 10.1021/acsomega.6c05008

**Published:** 2026-07-10

**Authors:** Md Ariful Islam, Md Mehedi Hassan, Md Tanvir Hossain, Jakir Hossain Ridoy, Ahasan Habib

**Affiliations:** † Department of Textile Engineering, Dhaka University of Engineering and Technology, Gazipur, Gazipur 1707, Bangladesh; ‡ Department of Textile Engineering, 307181 Bangladesh University of Business and Technology, Dhaka 1216, Bangladesh; § Department of Materials Science and Engineering, 3968 Michigan Technological University, Houghton, Michigan 49931, United States

## Abstract

Nowadays, cotton textiles with metal oxide nanoparticle functionality exhibit promising antibacterial applications; although many aspects have been tested, fabric comfort and moisture-related performance have not been sufficiently explored. In this study, cotton (twill) fabric has been functionalized by synthesizing ZnO nanoparticles (ZnO NPs) via a controlled exhaustion process followed by thermal fixation. The synthesized nanoparticles are characterized by Fourier transform infrared spectroscopy (FTIR) and X-ray diffraction (XRD), whereas scanning electron microscopy (SEM) proves the surface morphology. Antibacterial evaluation shows effective activity against *Staphylococcus aureus* and *Escherichia coli*, with a large inhibition zone (35–36 mm) and a ∼99% reduction in bacterial counts compared to uncoated cotton. The results of the moisture management test (MMT) indicate that the spreading and one-way transport capacities of liquid are measurable and fluctuate by 20–30% compared to the standard, suggesting a change in surface wettability after ZnO deposition. Fabric Touch Tester (FTT) analysis showed significant improvements in bending rigidity, surface friction, roughness, compression, and thermal comfort, with comfort parameters increasing by 10–25% when using the nanocoating. These results highlight the multifunctional benefits of ZnO functionalization for advanced hygienic textiles.

## Graphical abstract

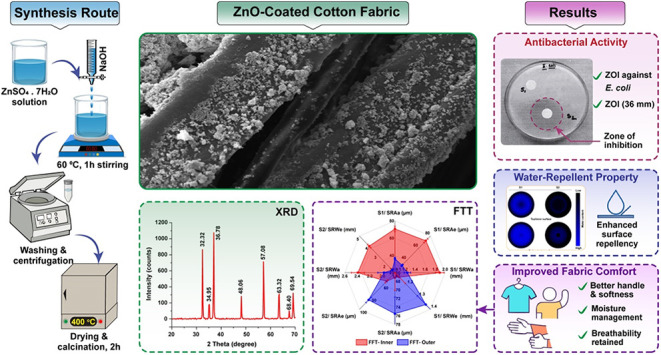

## 1 Introduction

Recently, nanoscience and nanotechnology have become part and parcel of the development of various types of advanced functional materials,^1^ enabling the development of materials with properties that are being addressed as a new industrial revolution in textiles.^2–4^ In recent years, the application of nanotechnology in textile materials has also been on the rise, particularly in the addition of extra functionalities such as antimicrobial activity,^5^ ultraviolet protection,^6^ self-cleaning behavior,^7^ wearability, and durability.^8^ These developments have broadened the application of textiles beyond traditional apparel to healthcare, hygiene, filtration, and biomedical applications, where performance from a functional perspective and comfort from the user’s perspective must be ensured simultaneously.^9,10^

Among natural textile fibers, cotton is still one of the most used substrates because of its availability, biodegradability, low cost, and wear comfort.^11,12^ The breathability and moisture absorbency, as inherent properties of cotton, make cotton particularly suitable as a fabric for garments and textiles where prolonged contact with skin is expected.^12,13^ However, the same characteristics that make inner surfaces highly hydrophobic and possess large specific surface areas also provide an ideal environment for the growth of microbes.^14^ The multiplication of bacteria on cotton textiles can result in unpleasant smells,^15^ fabric degradation, allergic reactions,^16^ and serious concerns about hygiene,^17^ which is particularly critical in biomedical and healthcare-related applications.^18^

The rising incidence of bacterial infections and the microorganisms that cause them developing resistance to traditional antimicrobial agents has only added to the need for effective antibacterial materials.^19,20^ In this context, textiles that can inhibit the growth of microorganisms without the use of conventional antibiotics have attracted a lot of attention.^21^ Nanoparticles, especially metal and metal oxide nanoparticles, have become promising antimicrobial agents,^22^ as a result of their broad-spectrum activity and a low probability of the development of resistance.^23^ Their antibacterial activity is commonly explained by several mechanisms, such as the disruption of bacterial cell membranes,^24^ the induction of reactive oxygen species (ROS),^25^ and the imbalance of intracellular metabolism.^26^

Recent developments in the area of advanced functionalization of textiles have generated considerable research interest in metal and metal oxide nanoparticles for their multifunctional properties and excellent antimicrobial activity.^27^ A considerable number of metal oxide nanomaterials have been studied to use them as antibacterial materials in textiles, including titanium dioxide (TiO_2_),^28^ copper oxide (CuO),^29^ magnesium oxide (MgO),^30^ silicon dioxide (SiO_2_),^31^ aluminum oxide (Al_2_O_3_),^32^ and zinc oxide (ZnO).^32^ TiO_2_ nanoparticles have a strong antibacterial performance, and they have very good photocatalytic and self-cleaning properties, but their antibacterial effect is, in general, triggered by UV light activation.^33^ Even though CuO nanoparticles are highly antimicrobial, they have higher cytotoxicity and can cause color change of textile substrates, which limits practical biomedical application.^34^ While the former (MgO nanoparticles) provide moderate antibacterial properties but with relatively low durability,^35^ the latter (SiO_2_ nanoparticles) are primarily applied to enhance surface roughness and hydrophobicity, but not as direct antibacterial agents.^36^ On the other hand, among all types of inorganic antimicrobial nanomaterials, ZnO nanoparticles have several unique properties, such as broad-spectrum antibacterial activity,^37,38^ UV protection, chemical stability, biocompatibility, low toxicity, and cost-effectiveness, which set them apart from other materials. Moreover, ZnO nanoparticles have ROS-generating abilities, the ability to release Zn^2+^ ions, and direct interaction with bacterial cell membranes,^39^ which results in the increased antimicrobial effect against both Gram-positive and Gram-negative bacteria, which was proved in numerous studies.^39,40^ For these reasons, ZnO nanoparticles are regarded as one of the most promising inorganic nanomaterials for a range of multifunctional applications in cosmetics,^41^ pharmaceuticals, food-related products, especially for wearable healthcare and biomedical products,^42^ which has simultaneously made it an antibacterial material useful for antibacterial textile development.^43^

Despite these advantages, the practical application of ZnO nanoparticles on cotton textiles is difficult. A recurring limitation reported in the literature is adequate adhesion of the nanoparticle to the fiber surface, which may result in poor durability during washing and poor functional durability.^43^ In addition, nanoparticle agglomeration and nonuniform surface coverage can affect efficiency, fabric quality, and antibacterial activity. Importantly, there are several reports that ZnO nanoparticles negatively affect the mechanical and handling characteristics of cotton fabrics, such as lowering the bending and tearing resistances, the abrasion resistance, and the flexibility.^44^ These drawbacks are serious obstacles to the extensive use of ZnO-functionalized cotton textiles.

In addition to mechanical durability, fabric comfort is an important issue for wearable and biomedical textiles. Comfort in itself is a multidimensional property and is characterized by a collection of factors that include bending behavior, on-surface friction, compression response, thermal properties, and moisture management performance.^45^ These properties can be significantly altered by surface modification with nanoparticles, increasing or decreasing the stiffness, roughness, and moisture transport. Thus, the antibacterial activity test is not a rigorous assessment of practical textiles to be used in practice.

Traditional mechanical tests, including assessments of tensile and tearing strength, are insufficient to capture the finer details of tactile and handling characteristics that influence the wearer’s perception. In this regard, the Fabric Touch Tester (FTT) is a more holistic and objective method because it simultaneously measures the bending, surface, compression, and thermal responses.^46^ The FTT-derived indices give information as to the fabric mobility, smoothness, softness, and even warmth and allow for a more realistic assessment of comfort-related performance.^47,48^ However, despite its relevance, the application of FTT analysis to the study of ZnO-functionalized cotton textiles remains limited. Another fundamental feature concerning antibacterial and biomedical textiles is the moisture management behavior. Efficient liquid absorption, spreading, and transport are not only important for wearer comfort but also for hygienic performance, limiting the occurrence of localized moisture buildup, which can contribute to bacterial growth.^46^ Nanoparticle deposition can alter the wetting characteristics and capillary flow path on the fabric surface, making the assessment of moisture management a necessary part of the functional appraisal of the textiles.

In this study, ZnO nanoparticles are synthesized from ZnSO_4_·7H_2_O using a controlled exhaustion-based treatment and thermal fixation method for the functionalization of cotton fabric to realize the stable anchoring of nanoparticles to the fabric. Clear structure–property–function relationships were established using a comprehensive characterization strategy.

Fourier transform infrared spectroscopy (FTIR), X-ray diffraction (XRD), and scanning electron microscopy (SEM) were used to investigate chemical interactions and crystalline properties and the surface morphology and distribution of nanoparticles, respectively. The moisture management behavior was evaluated for liquid transport, and the antibacterial activity was assessed. More importantly, the zone of fabric comfort and mobility was included in the FTT analysis. Using the derived indices of FTT comfort as correlates of surface modification, moisture behavior, and antibacterial performance, this study aims to help bridge a significant gap in existing studies addressing the antimicrobial functionality and comfort in textiles. The results provide a systematic pathway to understanding the effects of ZnO nanoparticle functionalization on cotton fabric performance and offer practical insights into the development of antibacterial textiles for biomedical and healthcare applications, where durability, hygiene, and comfort must coexist.

## 2 Experimental Design

### 2.1 Materials

A 100% cotton woven fabric was sourced from a local factory in Gazipur, Bangladesh. The fabric’s composition and specifications were provided by the supplier, as detailed in Table 1. Chemicals, including zinc sulfate heptahydrate (ZnSO_4_·7H_2_O, molecular weight 287.56 g/mol, 99% purity), sodium hydroxide (NaOH, MW 39.997 g/mol, 99% purity), ethanol (C_2_H_5_OH, MW 46.07 g/mol), hydrogen peroxide (H_2_O_2_), as well as sequestering, wetting, and leveling agents, were purchased locally at Hatkhola, Dhaka, Bangladesh. The chemicals and reagents used in this study were of analytical grade and were not further purified.

**Table 1:** Specification of 100% Cotton Fabric

fabric composition	cotton
fabric type	woven
fabric structure	twill
warp count	25
weft count	25
ends per inch (EPI)	125
picks per inch (PPI)	65
fabric GSM	164

Raw cotton is then washed, treated with bleach, and nanocoated to enable it to absorb and remain permanently white.

### 2.2 Preparation of ZnO Nanoparticles

The controlled precipitation process was carried out in an initially acidic medium to produce the ZnO nanoparticles. All chemical manipulations and solution mixing were performed in a laboratory fume hood to ensure adequate ventilation and to mitigate exposure of operators to corrosive NaOH fumes, aerosol droplets during dropwise addition, and any release of reaction byproducts.

ZnSO_4_·7H_2_O (0.2 M) was dissolved in deionized water to make an aqueous solution (Figure 1a). To the reaction, 25 mL of this precursor solution was diluted with 50 mL of deionized water, which gave an initial pH of about 5.6, which was measured before the addition of the alkaline reagent. The solution of NaOH (4.0 M) (25 mL) was added dropwise into the solution containing zinc at a constant rate of about 5 mL/min with constant magnetic stirring (∼600 rpm) (Figure 1b). This sequence of addition, in which the alkaline solution is slowly added to an initially acidic medium of zinc precursors, will characterize the acidic route of precipitation and enhance rapid supersaturation in the initial phase of the reaction (Figure 1c). This then leads to the production of a high density of nuclei that prefer the equiaxed ZnO nanoparticles. Table 2 lists all of the parameters.

**Figure 1:**
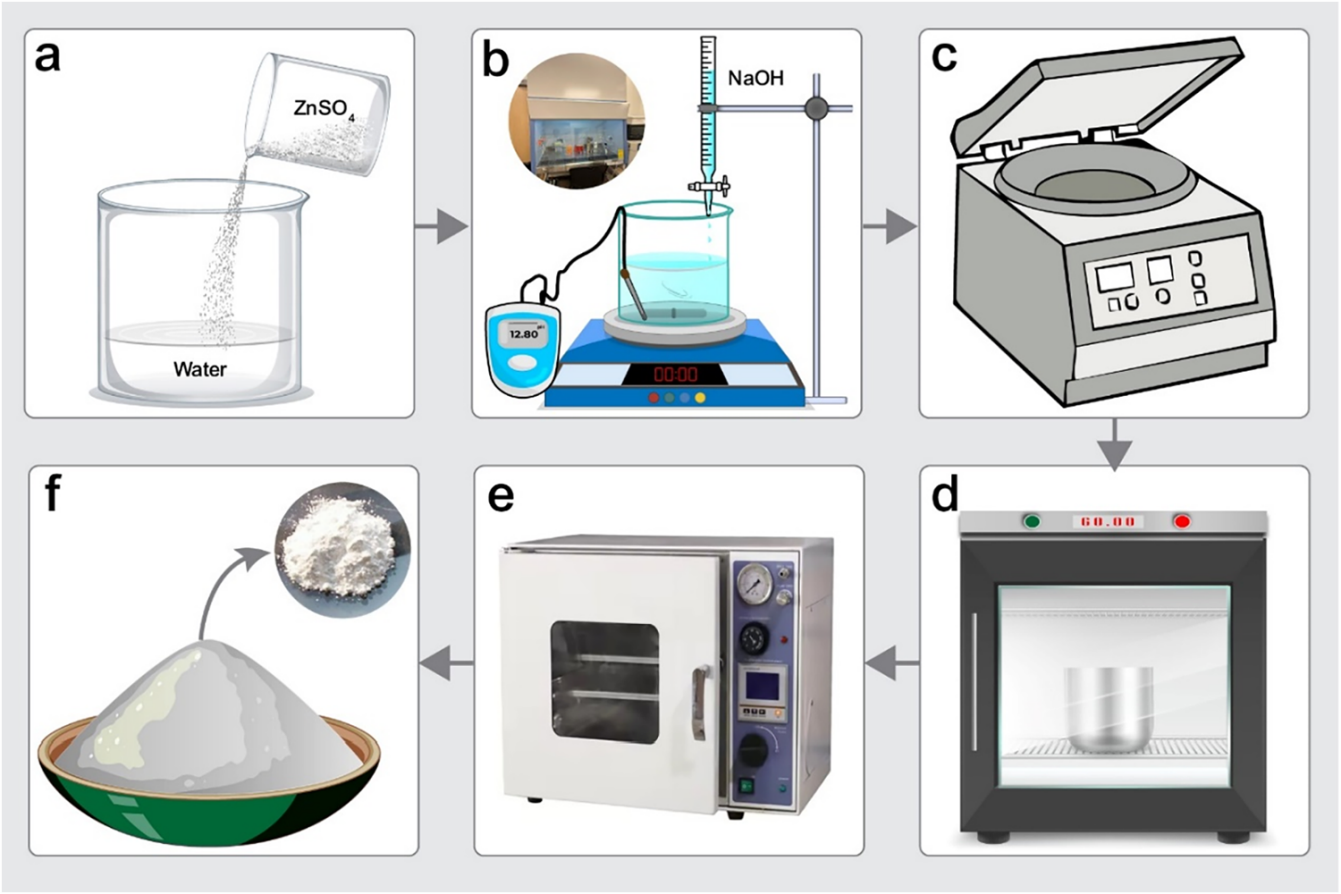
Synthesis process of ZnO nanoparticles: (a) mixing ZnSO_4_ with deionized water, (b) adding NaOH dropwise to the mixture, (c) centrifuging the solution, (d) drying at 60 °C, (e) calcination at 400 °C for 3 h, and (f) obtaining the synthesized ZnO nanoparticles.

**Table 2:** Experimental Conditions as well as the Characteristics of the Particles

parameter	value	parameter	value
synthesis method	acidic precipitation	final pH^b^	12.8
addition mode	NaOH added into Zn^2+^ solution	reaction temperature	60 °C
[OH^–^]/[Zn^2+^] molar ratio	20	reaction time	2 h
initial pH^a^	5.6	calcination condition	400 °C, 3 h

a The pH was first recorded prior to the addition of the NaOH.

b This was obtained after incubating the reaction mixture at 60 °C for a period of 2 h.

The reaction mixture was thoroughly mixed to obtain effective concentrations of approximately 0.05 M Zn^2+^ and 1.0 M OH^–^, and a molar ratio of [OH^–^]/[Zn^2+^] of 20. It was incubated at 60 °C for over 2 h, during which the pH rose to about 12.8 (after thermal treatment). The precipitate was centrifuged to separate the product and then the product was washed with deionized water until the product was free of ionic species and dried at 60 °C (Figure 1d). In order to increase the degree of crystallinity and complete conversion to ZnO, the dried product was further calcined in air at 400 °C for 3 h to form a fine white powder of ZnO (Figure 1e,f). In this case, Zn^2+^ ions are first converted into hydroxide-like intermediates, which are then converted to crystalline ZnO through thermal treatment.

ZnO forms under alkaline conditions through hydroxide-mediated reactions, where Zn^2+^ reacts with OH^–^ to create zinc hydroxide intermediates, which convert to ZnO during drying and calcination. The simplified as follows:

$$\begin{array}{l} Zn^{2+}+2OH^{-}\to Zn{\left(OH\right)}_{2}\\ Zn{\left(OH\right)}_{2}\to ZnO+H_{2}O↑ \end{array}$$

### 2.3 Applying ZnO Nanoparticles as a Coating on Cotton Fabric

Cotton fabric was coated with zinc oxide (ZnO) nanoparticles using an exhaustion method and subsequently subjected to a thermal fixation process. Previously synthesized ZnO nanoparticles were dispersed in an aqueous medium at 2% (pH 6.5–7.5), and the fabric was treated in a laboratory-scale dyeing machine at 80 °C for 20 min under continuous agitation to ensure uniform exhaustion and adsorption of nanoparticles onto the cotton fibers. Figure 2a shows the ZnO coating process via the pad-dry-cure route, in which the fabric was immersed in a ZnO dispersion and then squeezed.

**Figure 2:**
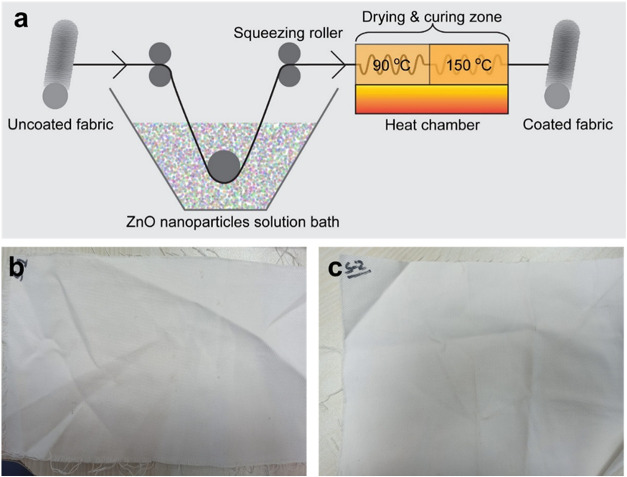
(a) Systematic application process of ZnO nanoparticles on cotton fabric, (b) uncoated fabric, and (c) ZnO-coated fabric.

After the exhaustion process, the coated fabric was thermally dried at 90 °C (first heating chamber) and then cured at 150 °C for 5 min in a curing oven to promote the stable fixation of ZnO nanoparticles via thermally induced interfacial interactions with the cellulose matrix. The cured samples were subsequently washed to remove loosely bound nanoparticles, then dried in a fabric dryer (Figure 2c). Uncoated cotton fabric was processed under identical drying and curing conditions to maintain a consistent processing history for comparison (Figure 2b).

### 2.4 Characterizations

To evaluate the impact of ZnO functionalization, we characterized the uncoated and ZnO-coated cotton fabrics. The chemical interactions, crystalline structure, and surface morphology were studied using FTIR, XRD, and SEM, whereas the moisture, antibacterial, and comfort-related properties were investigated using the Moisture Management Tester (MMT), antibacterial assays, and the Fabric Touch Tester (FTT) to analyze the antibacterial and biomedical use of textiles.

#### 2.4.1 Fourier Transform Infrared Spectroscopy (FTIR)

FTIR was also used to examine the functional groups of the cotton fabric and determine the potential interactions between cellulose and ZnO nanoparticles (IR Prestige-21, Shimadzu Corporation, Japan). IR measurements had a spectral resolution of 4 cm^–1^ and a spectral range of 400–4000 cm^–1^. Such an analysis was carried out in order to identify common absorption bands and discuss possible interactions between the fabric substrate and ZnO nanoparticles.

#### 2.4.2 X-ray Diffraction (XRD)

The crystallinity of the ZnO nanoparticles was examined, and the conversion of cotton crystallinity when in the presence of ZnO was determined using XRD. Knowing the structural integrity and phase properties of the functionalized fabric is important. An X-ray diffractometer (D/MAX 2550PC, Japan) was used to investigate the crystal structure of the samples, with an accelerating voltage of 40 kV and a current of 30 mA using Cu Kα radiation (λ = 0.15406 nm). The patterns of the diffraction were scanned at a rate of 1°/min over a range of 2θ of 5–60°. The samples were analyzed under the Segal method, which estimated the crystalline index (CrI) based on the ratio between crystalline and amorphous peak intensities of diffraction (eq 1).

1 $$\mathrm{CrI}\;\left(\%\right)=\frac{I_{200}-I_{\mathrm{am}}}{I_{200}}\times 100$$

where *I*_200_ represents the max intensity of the (200) lattice diffraction peak at 2θ = 22.5°, and *I*_am_ denotes the intensity of the amorphous peak at 2θ = 18°.

#### 2.4.3 Moisture Management Properties

The moisture management tester (MMT, model: M290, SDL Atlas, U.K.) was used to determine the impact of the ZnO coating on the liquid absorption, spreading, and transport behavior of the cotton fabric. The moisture management of the fabrics was measured by the AATCC 195-2009 method. The top and bottom fabrics were used to determine the time to wet, the rate of wetting, the maximum wetted radius (MWR), the spreading speed (SS), the one-directional capacity for transporting moisture, and the overall capacity to handle moisture. The tests were repeated a few times, and the average values have been reported.

#### 2.4.4 Antimicrobial Assay

The Kirby-Bauer disc diffusion technique was used to assess the antibacterial properties of the experimental fabrics against *Escherichia coli* (ATCC 29213) and *Staphylococcus aureus* (ATCC 25922).^49,50^ The tryptone soya agar (TSA) plate was used to grow new bacterial cultures. A single colony was transferred into tryptone soya broth and incubated overnight at 37 °C to obtain a bacterial suspension similar to a 0.5 McFarland standard (1.5 × 10^5^ CFU/mL). The suspension was then diluted with sterile normal saline in order to obtain the required working concentration. On agar plates, the specimen fabrics were inoculated and incubated at 37 °C for 24 h. The antimicrobial test was conducted by measuring the zone of inhibition (ZOI) around the fabric samples.

#### 2.4.5 Morphological Observation

The process of examining the surface morphology of uncoated and ZnO-coated cotton fabrics was conducted by scanning electron microscopy (SEM) (SU1510, Hitachi, Japan). SEM images (1000× and 5000×) were acquired at an accelerating voltage of 10 kV to visualize the fiber structure and the distribution of nanoparticles.

#### 2.4.6 Fabric Comfort Evaluation

The Fabric Touch Tester (FTT, SDL Atlas, model M293) was used to analyze changes in tactile, surface, compression, and thermal comfort due to the ZnO coating. Sample preparation before the tests: The samples were stored for 24 h at 20 ± 2 °C and 65 ± 4% relative humidity. The orientations of the two sides of the material and the major directions of the material were taken. The obtained indices were entered into a program known as FTT, which could predict key comfort properties, including smoothness, softness, and warmth.

## 3 Results and Discussion

### 3.1 FTIR Analysis

Figure 3 shows the FTIR spectra of both fresh cotton fabric (S1) and ZnO nanoparticle-coated cotton fabric (S2) within 4000–400 cm^–1^. S1 has a large absorption band with a peak at about 3327 cm^–1^ that is ascribed to the *K*-value (stretching vibrations) of the hydroxyl (−OH) groups in cellulose and the physically adsorbed moisture, which aligns with previous literature.^51^ The absorption band at approximately 1608 cm^–1^ is attributed to the bending vibration of H–O–H of the absorbed water molecules, whereas the peak at 1508 cm^–1^ is attributed to the C–H bending vibrations of the cellulose backbone. In addition, the typical band at 1080 cm^–1^ is due to C–O–C vibrations associated with the β-1,4-glycosidic bonds of cellulose, indicating that the native cotton structure was retained.

**Figure 3:**
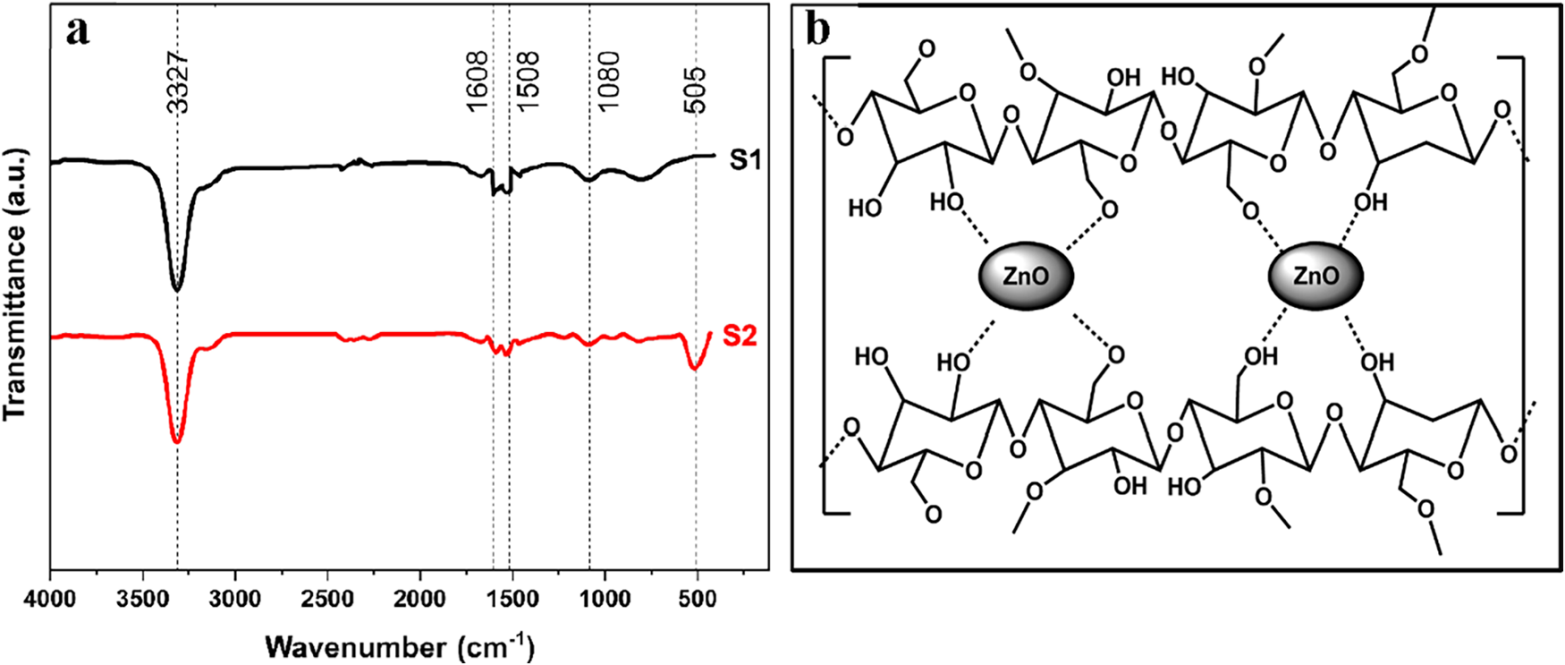
(a) FTIR spectrum of cotton (S1) and ZnO-coated cotton (S2), and (b) proposed chemical structure of ZnO-coated cotton fabric.

On the other hand, the FTIR spectrum of S2 in the sample of the ZnO nanoparticle-coated sample has the same size as major cellulose-related absorption bands, which means that the chemical structure of the cotton substrate is not altered after the surface functionalization (Figure 3a). It is important to note that the intensity of the O–H stretching band at 3327 cm^–1^ decreases and the band becomes slightly broader, which indicates that there are intense interfacial interactions between ZnO nanoparticles and hydroxyl groups of cellulose, which may be through hydrogen-bonding and coordination interactions. More so, a specific band of absorption is present at around 505 cm^–1^, which is specific to Zn–O vibration and confirms the effective attachment of ZnO nanoparticles on the surface of the cotton fiber.^52^ The coexistence of cellulose functional groups and Zn–O vibrations indicates the successful interfacial incorporation of ZnO nanoparticles between the cotton fibers, enabling durable surface behavior without altering the inherent structure of the textile substrate. From Figure 3b, ZnO nanoparticles react with hydroxyl groups of cellulose through hydrogen bonding and coordination interactions to increase adhesion to the cotton surface.^53^

### 3.2 X-ray Diffraction Analysis

The structural properties and phase purity of the synthesized ZnO nanoparticles were investigated using X-ray diffraction (XRD). The diffraction pattern (Figure 4) exhibits well-defined peaks at 2θ ≈ 32.32, 34.95, 36.78, 48.06, 57.08, 63.32, 68.40, and 69.54°, which is similar to previous studies.^54^ These observations are consistent with the (100), (002), (101), (102), (110), (103), (112), and (201) crystal planes of hexagonal wurtzite ZnO, which confirm successful growth of the required crystalline phase.^52^ The absence of any other peaks indicates that it is highly purified, with no impurities.

**Figure 4:**
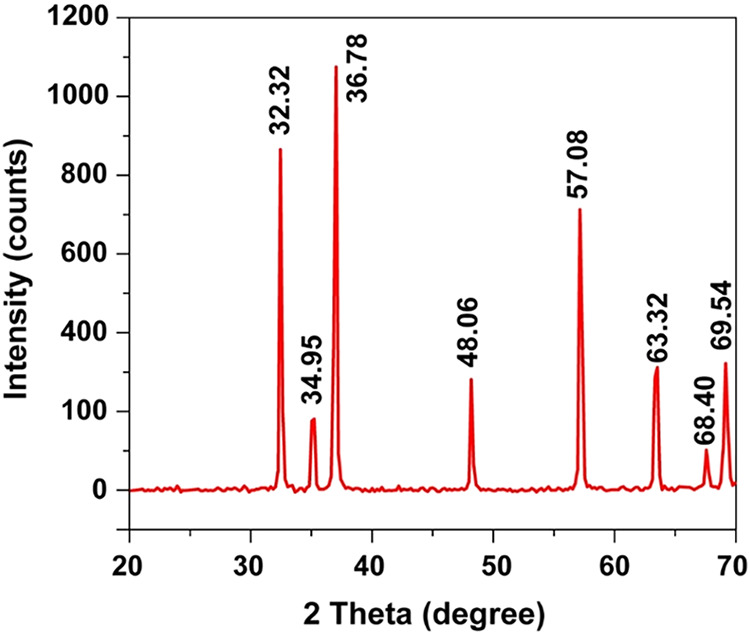
XRD diffraction patterns of synthesized ZnO nanoparticles.

The most intense diffraction peak is at 36.78°, corresponding to the (101) plane, indicating a preferred orientation along this crystallographic direction. The intense diffraction peaks are sharp and intense, implying high crystallinity, whereas the finite broadening of the peaks implies nanoscale crystallites.

The particle size (*D*) was estimated using the Scherrer eq 2:^51^

2 $$D=\frac{0.9\times \lambda }{\beta \times \cos\theta }$$

In which λ = 15,406 nm, β is the full-width-half-maximum (fwhm), in radians, and θ is the X-ray diffraction angle. The major diffraction peaks yielded crystallite sizes summarized in Table 3.

**Table 3:** Crystallite Size Calculated from XRD Peaks of ZnO NPs

2θ (deg)	fwhm (deg)	θ (deg)	β (rad)	crystallite size (nm)
32.32	0.168	16.16	0.00293	49.6
34.95	0.164	17.47	0.00286	50.7
36.78	0.187	18.39	0.00326	44.7
48.06	0.198	24.03	0.00345	44.0
57.08	0.215	28.54	0.00375	42.3
63.32	0.208	31.66	0.00363	43.8
68.40	0.159	34.20	0.00277	56.3
69.54	0.227	34.77	0.00396	39.8

The average crystallite size was calculated to be ∼46.4 nm, confirming the development of ZnO nanoparticles at the nanoscale (less than 100 nm). The variation in crystallite size across crystallographic planes indicates anisotropic crystal growth, a common feature of ZnO nanostructures. Moreover, the values of fwhm are relatively low, which means that crystallinity is good and that there is a minimum number of lattice defects.

### 3.3 Moisture Management Evaluation

The moisture management tester (MMT) was used to assess the moisture management behavior of uncoated cotton fabric (S1) and ZnO nanoparticle-coated cotton fabric (S2) when assessing the wetting time (WT), absorption rate (AR), maximum wetted radius (MWR), spreading speed (SS), and one-way transport capability (OWTC) on the surfaces of the two fabrics.

The cotton (S1) wetted rapidly on both fabric surfaces, with wetting times of 1.97 s (top) and 1.50 s (bottom). The wetting times of the ZnO-coated top and bottom surfaces were 5.80 and 13.39 s, respectively, corresponding to increases of ∼194 and ∼793%, respectively (Figure 5a). This considerable delay indicates a strong decrease in surface wettability.^55^ Similarly, the absorption rate through the liquid decreased significantly after coating. The absorption rate at the top surface decreased from 11.57 to 6.04, and at the bottom surface, from 29.96 to 4.14. The spreading behavior was also inhibited, with the maximum wetted radius reducing from 20 mm (S1) to 5 mm (S2) on the top surface and to zero on the bottom surface of S2. The most significant drop in transport capability occurred from S1 to S2, from 213.22 to 0.46, implying a decrease of over 99%. This is due to the deposition of ZnO nanoparticles that partially cover hydrophilic groups on cellulose and narrow capillary channels, thereby reducing moisture movement through the fabric. Figure 5b shows that, on both surfaces, S1 displays increased and regular water content, which demonstrates effective moisture spreading due to porosity and reduced fabric surface tension, which is why its moisture transport behavior is better than that of coated fabric. With respect to modified water content and limited spreading, S2 has less water content, confirming that it reduces moisture uptake due to the porosity of the fabric and the effects of nanoparticles. The results show that the ZnO-coated cotton fabric was shown to have a water-repellent (hydrophobic) nature because of the higher surface roughness and less porous structure caused by the deposition of nanoparticles.

**Figure 5:**
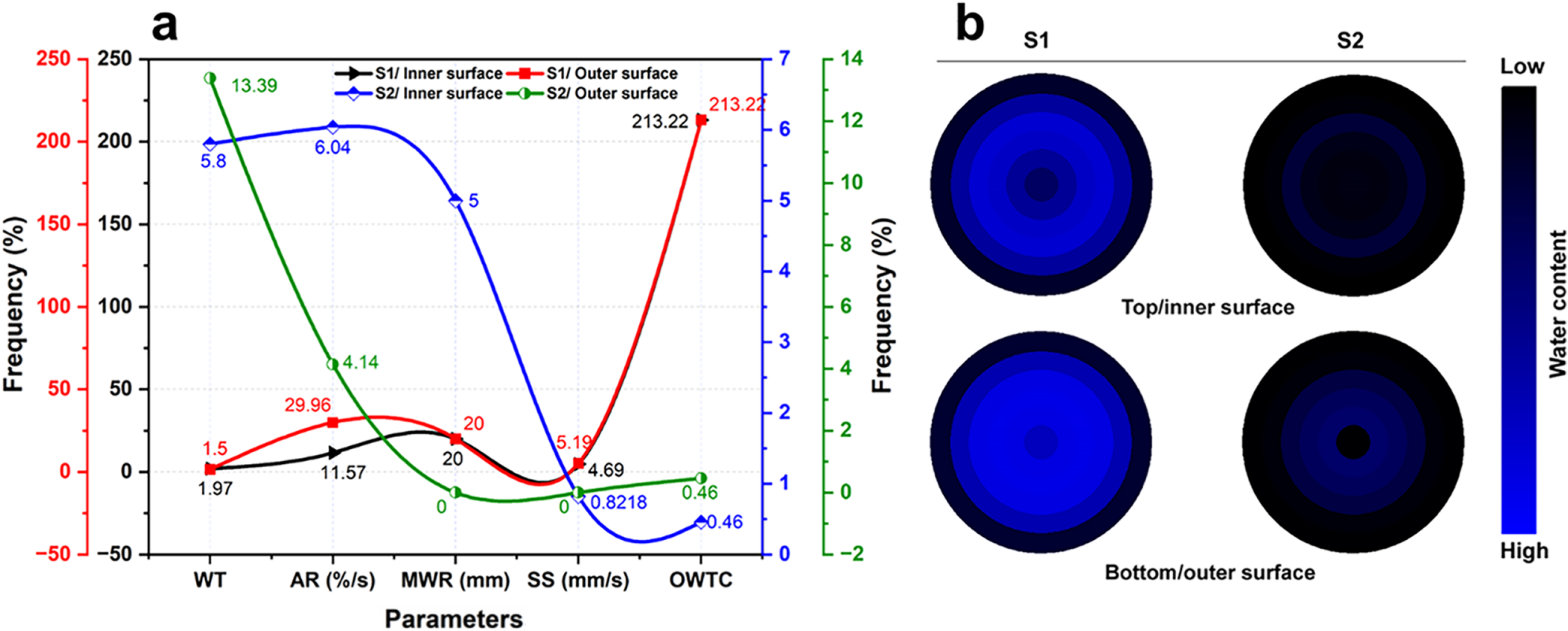
Moisture behavior of uncoated and coated samples; (a) moisture management properties curve and (b) water content vs time diagram.

Although the absorption rate and wetted radius were slightly decreased for both the untreated and treated fabrics after coating, the moisture management performance of the ZnO-coated fabric remained acceptable for clothing intended for wearing as textiles. Another possible mechanism for improving moisture management and wearer comfort of the ZnO-treated fabric under practical conditions is the controlled alteration of the hydrophilic/hydrophobic balance achieved by depositing ZnO on the fabric’s surface. Therefore, the cotton fabric loaded with ZnO nanoparticles showed good moisture management properties with enhanced antibacterial properties, which can be considered a potential application in multifunctional protective and healthcare textiles.

### 3.4 Antibacterial Activity

A qualitative disk diffusion assay of pristine cotton fabric (S1) and ZnO nanoparticle-coated cotton fabric (S2) against *E. coli* (Gram-negative) and *S. aureus* (Gram-positive) was used to test the antibacterial activity. The bacterial suspensions were formulated at 0.5 × 10^6^ CFUs/mL, and the tests were carried out on tryptic soy agar (TSA) plates under standard incubation conditions.

As shown in Figure 6a, the uncoated cotton (S1) exhibits no observable zone of inhibition (ZOI) against either of the two bacterial strains, which proves that untreated cotton does not have built-in antibacterial properties. In contrast, the ZnO-coated cotton nanoparticle (S2) showed strong antibacterial activity against both microorganisms (Figure 6b). *E. coli* was inhibited, producing a clear inhibition zone with a diameter of 36 mm, whereas a ZOI of 35 mm was produced against *S. aureus*.^1,56^ The effective antibacterial properties of the ZnO nanocoating are confirmed by the formation of well-defined areas of inhibition around S2. There was a minor variation in the antibacterial response of the two bacteria. In particular, the inhibition zone against *E. coli* was about 2.9% larger than that against *S. aureus*, suggesting slightly greater susceptibility to *E. coli*.

**Figure 6:**
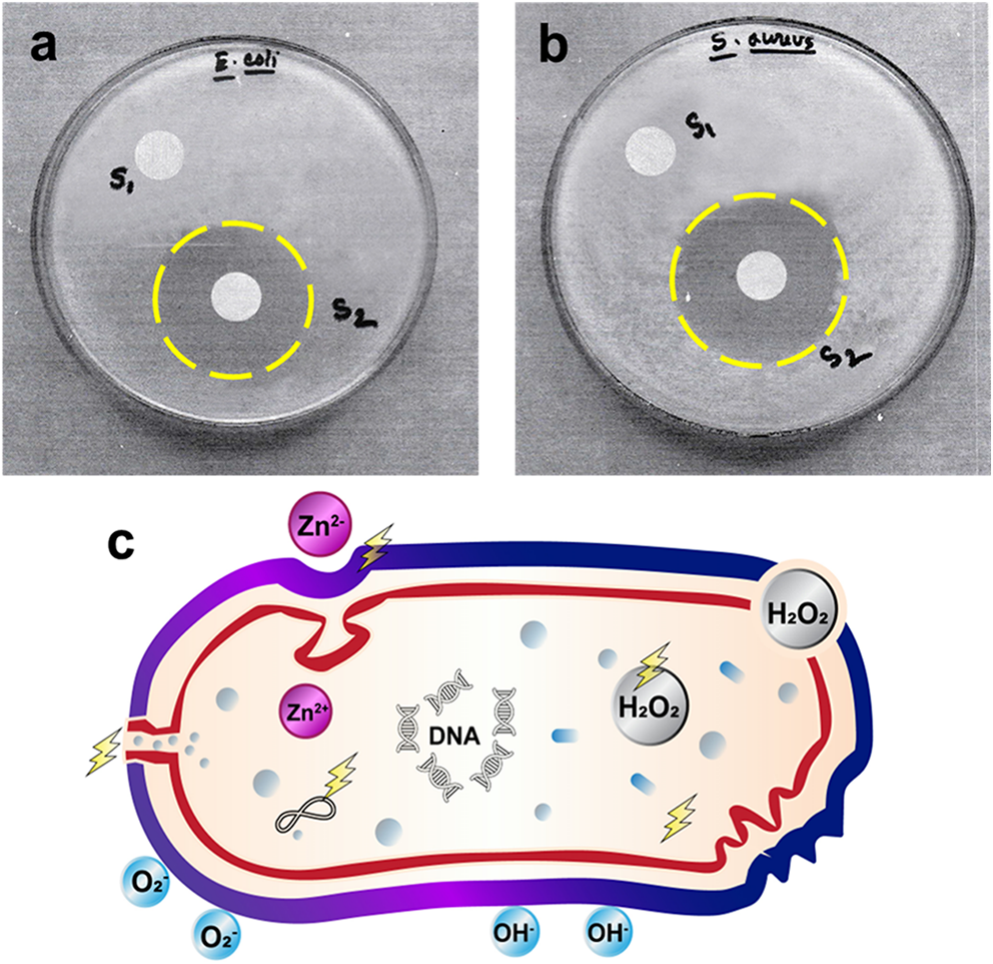
Antimicrobial activity test by disc diffusion method on cotton fabric against (a) *E. coli*, (b) *S. aureus* bacteria, and (c) antibacterial activity mechanism of ZnO nanoparticles.

Here, the antibacterial activity of the ZnO-coated cotton material is greatly increased by the presence of a binder. Compared with our previous results, in which ZnO-treated cotton exhibited lower antibacterial activity and a short wash life, the current results meet the performance standards for antibacterial textile materials. Finally, these findings confirm that the ZnO nanocoating effectively converts cotton fabric into a potent antibacterial material without compromising the biological inactivity of the uncoated substrate. Here, the mechanisms by which ZnO nanoparticles kill bacteria include the production of Reactive Oxygen Species (ROS) that are toxic to bacteria, the release of antimicrobial Zn^2+^ ions, and the disruption of the bacterial cell membrane, which are similar to those reported in previous studies.^57^ This is because they are able to penetrate the microorganism (the smallest in size) and then stop all life-sustaining cellular processes (Figure 6c).

$${O_{2}+e^{-}\to O}_{2}^{-}$$

H2O→OH•

It also damages the cell membrane and releases Zn^2+^ ions, thereby disrupting key metabolic processes. ZnO demonstrated comparable antibacterial activity in this study, and a relatively larger ZOI was observed.

### 3.5 Analysis of Surface Morphology

The surface morphology of the cotton fabric and ZnO nanoparticle-coated cotton fabric was evaluated using a scanning electron microscope (SEM) to assess the effect of ZnO deposition on the surface of the cotton fiber. Representative SEM micrographs are shown in Figure 7 at different magnifications (2 and 10 μm).

**Figure 7:**
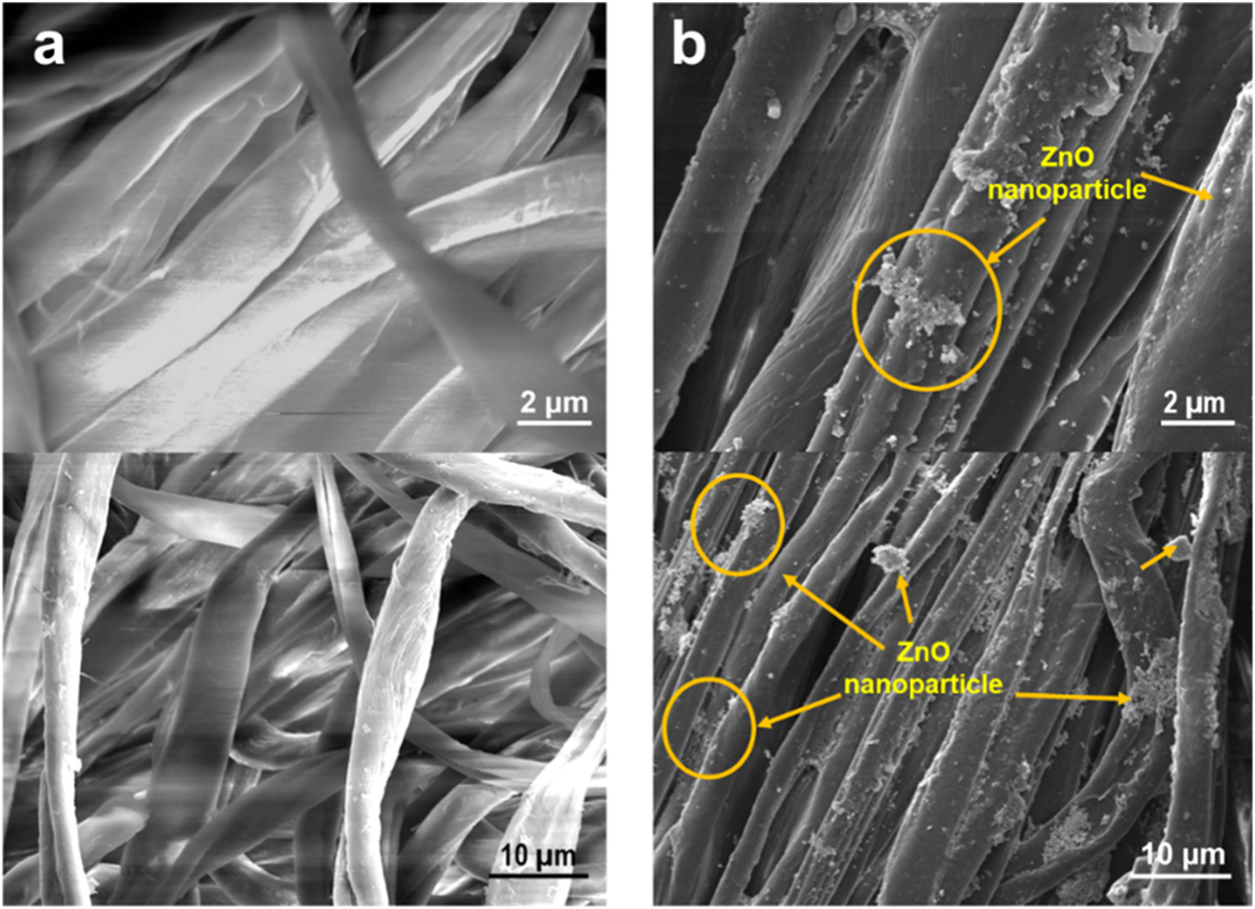
Surface morphology of the (a) cotton fabric and (b) ZnO nanocoated cotton fabric.

The SEM images of cotton fabric (S1) show smooth, clean, and well-defined fiber surfaces, free of particulate matter and surface irregularities (Figure 7a). At higher magnification, the normal longitudinal grooves of the native cotton fibers are clearly visible, whereas the lower-magnification image confirms the loosely packed fibrous network, as well as the unmodified nature of the cotton substrate.

In contrast, the ZnO nanoparticle coating on the S2 sample has significantly changed the surface morphology, which is consistent with prior studies.^52^ Numerous granular, irregularly shaped particles can be seen on the fiber surfaces clearly, as shown in Figure 7b. These surface features do not appear in S1 and were attributed to the successful deposition of ZnO nanoparticles on the cotton fibers. At higher magnification, the nanoparticles are seen tightly bonded to the fiber surface in discrete clusters rather than forming a continuous film, whereas at lower magnification, the distribution of nanoparticles along the fiber length is visible. Importantly, no obvious fiber damage, cracking, or structural collapse is observed after ZnO coating, indicating that no obvious changes to the intrinsic fibrous structure of cotton are observed during the coating process. Moreover, the SEM analysis provides evidence of good surface functionalization of cotton fibers with ZnO nanoparticles, with clear morphological evidence of nanoparticle attachment without jeopardizing the structural integrity of the textile substrate.

### 3.6 Analysis Using Fabric Touch Tester (FTT)

The tactile and mechanical properties of cotton fabric and ZnO nanoparticle-coated cotton fabric were tested using a Fabric Touch Tester (FTT). Measurements were made along the two main directions, where “a” and “e” are the warp and weft directions, respectively, in order to consider the anisotropy of the fabric. Both the inner and outer surfaces were analyzed to evaluate changes in the surface dependence of the ZnO coating. The FTT outcomes are explained with regard to bending, surface friction, surface roughness, and compression behavior.

#### 3.6.1 Bending Behavior

The bending behavior of cotton and ZnO-coated cotton nanoparticles was studied in terms of the bending average rigidity (BAR) and bending work (BW). For the inner surface, the bending rigidity of the warp direction (BARa) increased significantly from 198.65 (S1) to 335.12 gf/mm/rad in S2, which was approximately increased by 68.7%, showing an increase in the resistance to bending on the warp direction after ZnO coating (Figure 8a). In contrast, the rigidity in the weft direction (BARe) decreased significantly (60.4%, from 668.17 to 264.78 gf/mm/rad), which might also mean a redistribution of the bending stiffness between the fabric directions (Figure 8a). Such variability is caused by the difference in the deposition of nanoparticles and structural anisotropy in woven cotton structures, with warp yarns generally being more tensioned and more sensitive to surface stiffening.

**Figure 8:**
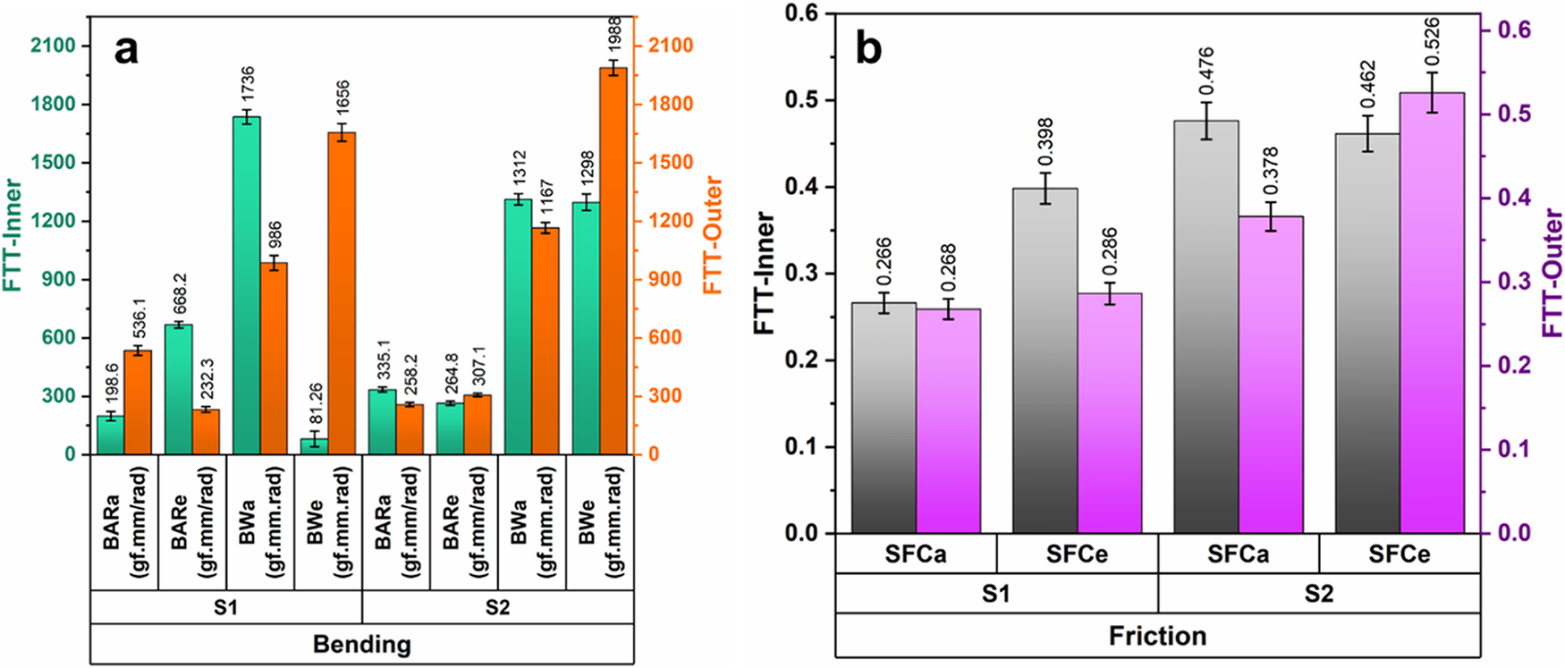
Mechanical touch behavior of samples: (a) bending and (b) friction behavior.

Bending work values also supported this trend. On the inner surface, BWa decreased by 24.4%, whereas BWe drastically increased owing to restricted yarn mobility obtained by surface-bound nanoparticles. For the outer surface, BARa decreased by 51.8%, while BARe increased by 32.2%, which led to a balanced bending response (Figure 8a). As a whole, the measured fluctuations are an integral combination of fabric construction and the stiffening of the surface by ZnO, resulting in less bending anisotropy and a more homogeneous mechanical response without the excess of flexibility loss.

#### 3.6.2 Friction Characteristics of the Surface

Surface friction behavior was quantified using the surface friction coefficient (SFC) to assess tactile resistance at the slide contact. Cotton (S1) showed quite low friction values on the inner and outer surfaces with SFCa values of about 0.27–0.29, which is an expression of the smoothness of untreated cellulose fibers (Figure 8b). After ZnO coating, a strong enhancement of the surface friction could be observed for S2. On the inner surface, SFCa increased from 0.27 to 0.48 (77.8%) and SFCe increased by 15.0%. On the outer surface, the increase in friction was even more substantial, and SFCa and SFCe increased by 40.7 and 82.8%, respectively (Figure 8b).

These fluctuations were mostly determined by the heterogeneous distribution of ZnO nanoparticles over the fabric surface. Nanoparticle clusters act like microasperities, increasing contact resistance during sliding, especially on the outer surface, where exposure to the coating is greater. The larger increase along the weft direction suggests a directional difference in the yarn exposure and surface topology. Despite this rise in friction, the SFC values remain within a moderate range, indicating that the ZnO coating improves functional surface roughness without making the fabric too harsh to the touch.

#### 3.6.3 Roughness Characteristics of the Surface

Surface roughness of both the warp and weft surfaces of cotton (S1) and the ZnO nanoparticle-coated cotton (S2) were analyzed using surface roughness amplitude (SRA) and surface roughness wavelength (SRW) of the two fabric surfaces. For the inner surface, a slight decrease in roughness amplitude was observed following ZnO coating. In particular, SRAa declined to 67.14 μm, about 5.0% lower than 70.64 μm, and SRAe declined much more significantly (from 72.27 to 54.17 μm), by about 25.0% (Figure 9a). This reduction indicates some smoothing by filling the microvalleys on the surfaces with nanoparticles. This trend, however, is accompanied by a notable change in roughness wavelength, with SRWa and SRWe improving by 26.5% (1.89–2.39 mm) and 242.1% (1.14–3.90 mm), respectively, indicating that broader, more widely spaced surface features have formed (Figure 9a).

**Figure 9:**
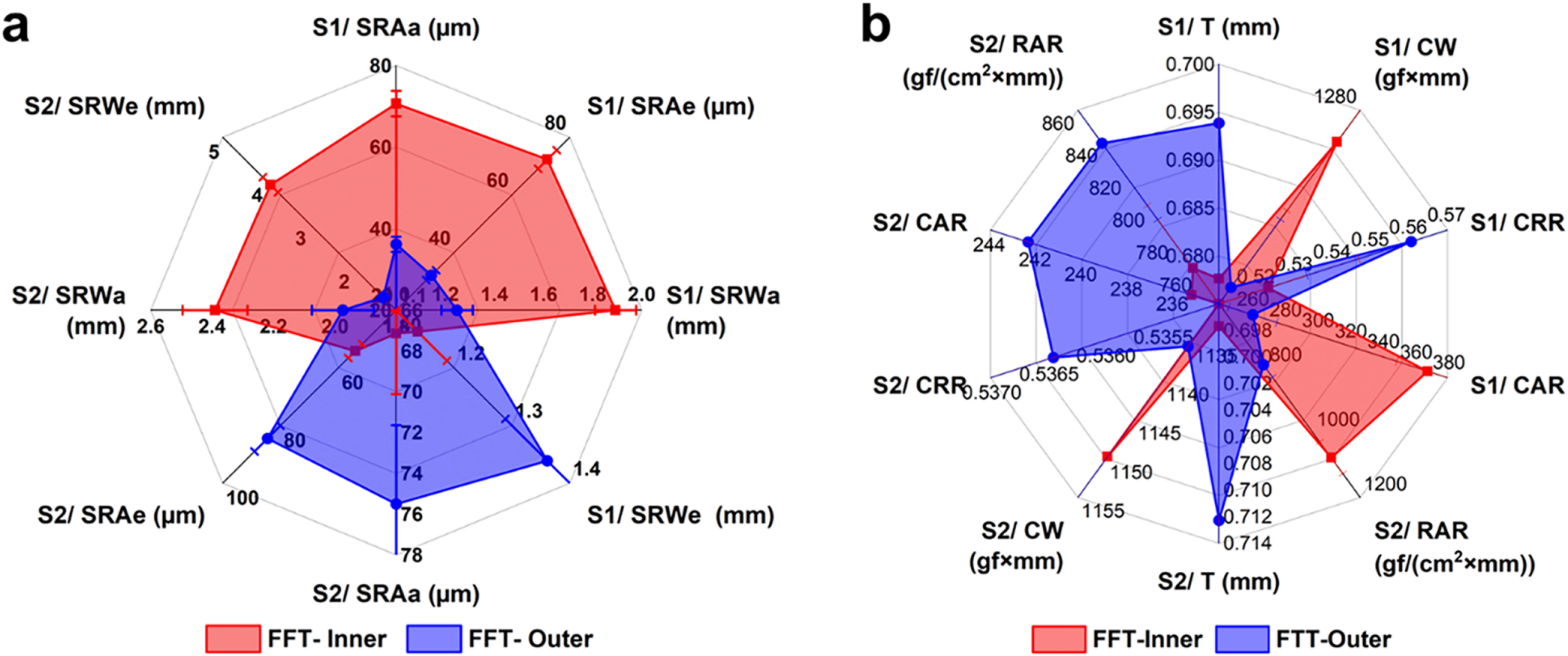
Fabric touch behavior (a) surface roughness and (b) compression behavior.

On the other hand, the surface roughness increases significantly with a ZnO coating on its outer surface. The SRAa rose to 75.50 μm with an increment of 108.6% (36.20 μm), and SRAe rose by 162.9% (32.15–84.52 μm) (Figure 9a). The SRW values at the outer surface also improved, with SRWa increasing by 57.6%, and SRWe increasing by only a small value of 4.4%. These variations are attributed to the uneven clustering of ZnO nanoparticles on the exposed fiber surface, and these findings are consistent with SEM observations.

#### 3.6.4 Compression Behavior

The compression behavior of pure cotton (S1) and ZnO nanoparticle-coated cotton (S2) was examined in terms of thickness (T), compression work (CW), compression average rigidity (CAR), compression recovery rate (CRR), and recovery average rigidity (RAR). After ZnO coating, the fabric thickness increased slightly on both surfaces: by 2.9% on the interior surface (0.68–0.70 mm) and by 2.9% on the exterior surface (0.69–0.71 mm), indicating the presence of an additional layer of nanoparticles (Figure 9b).

CW declined by 8.0% (1250.27–1149.74 gf mm) on the inside surface, while CAR declined by 36.1% (367.77–235.20), indicating improved compressibility after coating. At the same time, the CRR rose by 3.9%, indicating that elastic recovery was maintained to a large extent. RAR, on the other hand, was lower at 29.2%, which indicated decreased force during unloading. On the outside, CW was marginally up, 1.8%, and CAR fell by 7.0%. CRR was reduced moderately by 3.6%, whereas RAR rose by 6.4%, indicating a shift toward recovery behavior in the fabric surfaces. In general, compression tests show that the ZnO coating alters the near-surface compressive response and preserves sufficient bulk resilience of the fabric. The ZnO nanoparticles coated on cotton fabric are highly broad-spectrum antibacterial, without affecting the textile’s structural integrity. The surface modification also introduces minimal alterations to moisture and tactile properties, but its use is acceptable in biomedical applications. The cloth is thus suitable as an antibacterial, biomedical, and healthcare textile.

In addition, the Supporting Information (Figures S1 and S2) includes analyses of thermal comfort behavior and primary sensory indices. The results indicated that thermal transport, smoothness, and softness were only slightly affected by the ZnO nanoparticle coating with low wear comfort. The results also confirmed the feasibility of using ZnO-functionalized cotton fabrics as multifunctional wearable textiles.

## 4 Comparative Studies with Literature

The comparative analysis shows that the antibacterial, antioxidant, antifungal, and biocompatibility properties vary from one metal oxide NP to another (Table 4). TiO_2_ and CuO nanoparticles have excellent antibacterial properties, with TiO_2_ requiring UV activation and CuO exhibiting a relatively high toxicity. The nanoparticles of MgO, SiO_2_, and Al_2_O_3_ offer good biocompatibility but comparatively low multifunctional performance. On the other hand, ZnO nanoparticles exhibit a relatively good combination of broad-spectrum antibacterial activity, moderate antioxidant and antifungal activity, low toxicity, and compatibility with textiles. Moreover, the results show that cotton fabrics with a ZnO coating exhibit enhanced antibacterial properties without compromising moisture management and wear comfort. Hence, ZnO nanoparticles have been regarded as one of the most promising nanomaterials for use in a multifunctional approach to biomedical and healthcare textile applications.

**Table 4:** Comparative Analysis of the Biological Performance of Various Types of Metal/Metal Oxide Nanoscale Particles

nanoparticle type	synthesis route	antibacterial activity	antioxidant activity	antifungal activity	cytotoxicity/biocompatibility	refs
TiO_2_ NPs	sol–gel	UV-assisted antibacterial	low	mild	good biocompatibility	^24^
CuO NPs	hydrothermal	strong antibacterial	moderate	moderate	relatively higher toxicity	^58^
CuO/ZnO nanocomposite	chemical synthesis	broad-spectrum antibacterial	moderate	moderate	acceptable	^59^
MgO NPs	sol–gel	moderate antibacterial	moderate	mild	excellent biocompatibility	^53^
SiO_2_ NPs	Stöber method	weak antibacterial	low	weak	highly biocompatible	^60^
Al_2_O_3_ NPs	sol–gel	mild antibacterial	low	weak	high biocompatibility	^3^
ZnO NPs	green synthesis	strong against *E. coli* and *S. aureus*	moderate	moderate	low toxicity/good biocompatibility	^61^
ZnO-NPs-coated cotton	chemical synthesis	strong against *E. coli* and *S. aureus*	moderate	mild	skin compatible	^55^
ZnO-NPs-coated cotton	chemical synthesis	strong against *E. coli* and *S. aureus* (ZOI: 35–36 mm)				this study
Bio-ZnO NPs	plant-mediated	strong antibacterial	high antioxidant	moderate	low cytotoxicity	^62^
chitosan–ZnO NPs	biopolymer-assisted	enhanced antibacterial	high	strong	excellent compatibility	^54^
Al-ZnO NPs	plant extract-mediated	broad-spectrum inhibition	high DPPH scavenging	moderate	low toxicity	^63,64^

## 5 Conclusions

In this study, ZnO nanoparticles were successfully synthesized and applied on cotton fabric by the exhaustion method, followed by thermal fixation to obtain stable surface functionalization. Synthesis and morphological studies confirmed the uniform deposition of nanoparticles on the cotton surface. This modification showed significant antibacterial activity, exhibiting a large zone of inhibition against bacteria, whereas the MMT revealed water-repellent properties due to an increased surface roughness after ZnO coating. Importantly, analysis of these using FTT revealed measurable changes in fabric comfort and mobility, demonstrating the effect of ZnO functionalization on the tactile and handling properties. Also, thermal comfort behavior and primary sensory results indicate good performance, as shown in the Supporting Information. By integrating the synthesis of ZnO nanoparticles with exceptional antibacterial, moisture management, and comfort properties, this work provides clear structure–property–function relationships for the functionalized cotton fabrics. The results provide practical advice for the design of antibacterial textiles for biomedical and healthcare applications, where hygiene, durability, and wearer comfort are key issues that must be balanced.

## Supplementary Material



## Notes

Thermal comfort behavior: thermal conductivity during compression (TCC), thermal conductivity during recovery (TCR), thermal maximum flux (*Q*-max); and primary sensory indices: smoothness, softness, warmness value, and supporting Figures S1 and S2 supporting the findings of this study (PDF)
