# Supplementary material for: ZnO-Functionalized Cotton Textiles with Enhanced Antibacterial Activity, Moisture Management, and Wear Comfort
Source: ACS Omega. 2026 Jul 10;11(29):44401–12. doi: 10.1021/acsomega.6c05008 (PMC13425481; doi:10.1021/acsomega.6c05008)
Supplement: Supplementary file 1 [file ao6c05008_si_001.pdf]

Supporting Information

ZnO-Functionalized Cotton Textiles with Enhanced Antibacterial Activity, Moisture Management, and Wear Comfort

Md. Ariful Islam[1], Md. Mehedi Hassan[2], Md Tanvir Hossain[3*], Jakir Hossain Ridoy[2], Ahasan Habib[1]

[1] Department of Textile Engineering, Dhaka University of Engineering and Technology, Gazipur, Gazipur 1707, Bangladesh

[2]Department of Textile Engineering, Bangladesh University of Business and Technology, Dhaka 1216, Bangladesh

[3]Department of Materials Science and Engineering, Michigan Technological University, Houghton, MI, 49931, USA

*Corresponding author's email: mhossa39@mtu.edu

1. Thermal comfort behavior

The thermal comfort behavior of untreated cotton fabric (S1) and ZnO nanoparticle-coated cotton fabric (S2) was assessed based on FTT parameters, thermal conductivity during compression (TCC), thermal conductivity during recovery (TCR), and thermal maximum flux (Qmax). The outcomes show that cotton fabrics treated with ZnO nanoparticle deposition affected both thermal transfer and tactile perception.

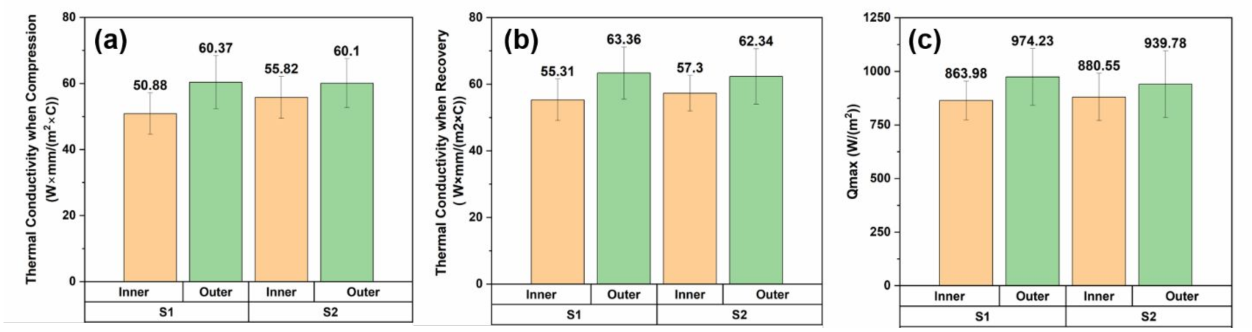

Figure S1. Thermal behavior of samples: (a) thermal conductivity when compression, (b) thermal conductivity when recovery, and (c) thermal maximum flux.

Figure S1(a) shows that the outer surface of S2 showed a slightly higher TCC value (55.82 W×mm/(m^2×C)) than the untreated cotton S1 (50.88 W×mm/(m^2×C)), which means that the heat transfer was slightly better during compression because of the presence of ZnO nanoparticles on the surface of cotton. Likewise, the TCR value of S2 increased slightly from 55.31 to 57.30 W×mm/(m^2×C) after deformation, indicating improved thermal recovery performance (**Fig. S1(b))**. In addition, the Qmax value of S2 also rose from 863.98 to 880.55 W/m2, which means the sensation of coolness during initial

skin contact was relatively higher for a higher Qmax value; a higher Qmax value corresponds to higher instantaneous heat transfer (**Fig. S1(c))**.

S2 also exhibited better thermal transport properties on the inside surface. On comparing the TCC and TCR values with S1, it was noted that the values slightly increased, whereas the Qmax value decreased from 974.23 to 939.78 W/m², with minor variation, implying that the ZnO coating maintained acceptable thermal comfort and thermal resistance.

2. Primary sensory indices

The main sensory indices showed moderate changes following ZnO functionalization, including smoothness, softness, warmth, and total hand value. The smoothness and softness of S2 decreased compared with untreated cotton; this could be attributed to the surface roughness and micro-asperities induced by the deposition of ZnO nanoparticles on both sides of the sample (**Fig. S2(a-d)**). This behavior is corroborated by SEM images, which show nanoparticle aggregates, resulting in a relatively rougher surface morphology. The outer surface, however, showed a small temperature increase (0.73), indicating that the ZnO coating did not significantly impact the fabric's thermal feel when worn.

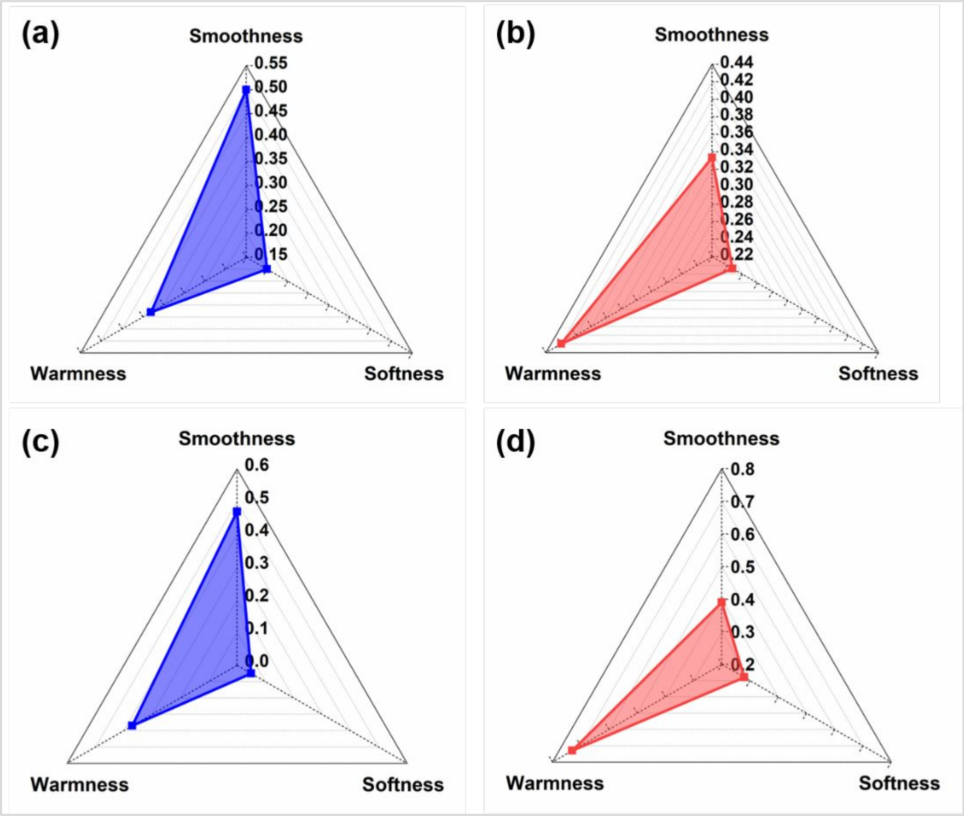

Figure S2. Primary Sensory Indices of samples (a) S1 inner, (b) S1 outer, (c) S2 inner, and (d) S2 outer radar chart.

The total hand value of the fabric slightly decreased after ZnO coating, but the fabric still maintained acceptable tactile comfort. The results reveal that the addition of ZnO nanoparticles has improved antibacterial properties without compromising thermal and sensory comfort. Thus, the multifunctionality of the ZnO-coated cotton fabric (S2) was better, given its enhanced thermal transport and antibacterial properties, with a slight reduction in tactile softness and smoothness. Based on the above findings, it was concluded that functionalization of ZnO nanoparticles is appropriate for biomedical and healthcare textiles intended for wearable use, where the wearer's hygiene and comfort are of critical importance.
